# Potency of pre–post treatment of coenzyme Q10 and melatonin supplement in ameliorating the impaired fatty acid profile in rodent model of autism

**DOI:** 10.3402/fnr.v60.28127

**Published:** 2016-03-03

**Authors:** Afaf El-Ansary, Mashael Al-Ghamdi, Ramesa Shafi Bhat, Sooad Al-daihan, Laila Al-Ayadhi

**Affiliations:** 1 Autism Research and Treatment Center, Riyadh, Saudi Arabia; 2 Shaik AL-Amodi Autism Research Chair, King Saud University, Riyadh, Saudi Arabia; 3 Biochemistry Department, Science College, King Saud University, Riyadh, Saudi Arabia; 4 Department of Physiology, Faculty of Medicine, King Saud University, Riyadh, Saudi Arabia

**Keywords:** brain, gas chromatography, neurotoxic, fatty acid, melatonin, coenzyme Q

## Abstract

**Background:**

Abnormalities in fatty acid metabolism and membrane fatty acid composition play a part in a wide range of neurodevelopmental and psychiatric disorders. Altered fatty acid homeostasis as a result of insufficient dietary supplementation, genetic defects, the function of enzymes involved in their metabolism, or mitochondrial dysfunction contributes to the development of autism.

**Objective:**

This study evaluates the association of altered brain lipid composition and neurotoxicity related to autism spectrum disorders in propionic acid (PA)–treated rats.

**Design:**

Forty-eight young male western albino rats were used in this study. They were grouped into six equal groups with eight rats in each. The first group received only phosphate buffered saline (control group). The second group received a neurotoxic dose of buffered PA (250 mg/kg body weight/day for 3 consecutive days). The third and fourth groups were intoxicated with PA as described above followed by treatment with either coenzyme Q (4.5 mg/kg body weight) or melatonin (10 mg/kg body weight) for 1 week (therapeutically treated groups). The fifth and sixth groups were administered both compounds for 1 week prior to PA (protected groups). Methyl esters of fatty acid were extracted with hexane, and the fatty acid composition of the extract was analyzed on a gas chromatography.

**Results:**

The obtained data proved that fatty acids are altered in brain tissue of PA-treated rats. All saturated fatty acids were increased while all unsaturated fatty acids were significantly decreased in the PA-treated group and relatively ameliorated in the pre–post melatonin and coenzyme Q groups.

**Conclusions:**

Melatonin and coenzyme Q were effective in restoring normal level of most of the impaired fatty acids in PA-intoxicated rats which could help suggest both as supplements to ameliorate the autistic features induced in rat pups.

The development of animal models of autism is one approach that could help identifying the mechanism by which autism develops in humans and testing the potency of selected supplements to ameliorate the impaired biomarkers related to it (1). Given the complexity of autism and its etiology, different approaches were developed to induce autistic features in rodents (1, 2). Our rodent model with autistic features was developed through orally administered neurotoxic dose of propionic acid (PA) (2). PA can change both brain and behavior in the laboratory rat in a manner that is consistent with symptoms of human autism spectrum disorder (ASD) (3). Thus, this model was designed to confirm the role of gut–brain axis in the etiology of autism, as orally administered PA was effective in inducing persistent brain toxicity and autistic features in rat pups (2).

The brain tissue of patients with autism show subtle developmental abnormalities, specifically in those areas concerned with language, facial expression, movement, and social behavior (3). Fatty acids are heterogeneous molecules that serve many roles, from providing cell structure to energy storage for cell signaling. The brain is one of the most lipid-enriched tissues in the human body, constituting 60% of dry weight (4). Over 20% of the dry weight of the brain is made up of polyunsaturated fatty acids, primarily docosahexaenoic acid and arachidonic acid, which are derived from essential fatty acids. Those fatty acids are concentrated in the neuronal membranous phospholipids, in the myelin sheath (5). Infants’ brains are small and undeveloped at birth and must incorporate fatty acids and cholesterol into the brain from circulation for it to develop properly (5). Some recent studies have suggested that fatty acid deficiency may be involved in autistic spectrum disorder (6). Also, there is growing evidence that fatty acid metabolism and abnormal membrane fatty acid composition may contribute to neurodevelopmental and psychiatric disorders (7). Reduced levels of polyunsaturated fatty acids have been associated with some childhood mental disorders, such as attention deficit hyperactivity disorder in boys (8, 9), severe deficits in reading, spelling, and auditory memory (5, 7), as well as dyslexia and developmental coordination disorder (10). Also, children with ASD have been shown to present significantly higher phospholipase A2 activity (11). In fact, evidence suggests that the instability observed in fatty acid levels may be caused by an increase in phospholipases A2 activity, perhaps in association with the high oxidative stress found in these patients (12). Recently, El-Ansary et al. (13) evaluated fatty acid profile in the plasma of 26 autistic children and 26 age-matched healthy children. The authors found increased levels of most saturated fatty acids, except for PA (due to high influx to brain), and reduced levels of most polyunsaturated fatty acids.

Several studies have shown a significant link between autism and mitochondrial problems. Additionally, autistic children's with mitochondrial dysfunction are more likely to have deficits in their ability to produce cellular energy due to abnormal fatty acid metabolism (14–17). Many recent studies have reported that nutritional supplements and/or antioxidants may be beneficial in some children with ASD who have mitochondrial dysfunction.

Reduced coenzyme Q10 as a component of the mitochondrial respiratory chain is effective by itself in reducing reactive oxygen species or by regenerating tocopherol as fat-soluble vitamin known to be lower in autistic patients (18). Decreased coenzyme Q10 concentration is associated with ASDs (19–21). On the contrary, insomnia as dysregulation of the melatonin pathway has been observed in many individuals with autism compared to typically developing controls. Treatment with coenzyme Q10, the only lipid antioxidant that is synthesized in mammals by all cells, has been reported to result in significant improvements in ASD symptoms in children (22, 23). Recently, another study reported improvements in children with ASDs using melatonin, although it was not reported if the children had concomitant mitochondrial dysfunction (24, 25).

Collectively, these studies suggest altered lipid metabolism may occur in ASDs. The brain tissue of PA-treated rats shows a number of ASD-linked neurochemical changes, including innate neuroinflammation, increased oxidative stress, glutathione depletion, and altered phospholipid/acyl carnitine profiles (1, 26). In this context, PA rodent model were used to examine whether there is any evidence for alterations in brain lipids associated with the occurrence of ASD. Pre–post treatments of coenzyme Q10 and melatonin supplement were also performed to determine if they play any role in altering brain lipid composition.

## Methods

### Animals

Male western albino rats (45–60 g, approximately 21 days old) were obtained from pharmacy college animal house at King Saud University and acclimated in our laboratory with standard conditions of temperature, 12-h dark/light cycle and were given free access to tap water and standard laboratory chow. After 1 week, the rats were divided into six groups (eight rats in each group), namely the control group in which animals were fed normal diet during the experimental period; second, the PA-treated rats that received 250 mg/kg body weight/day for 3 days, in order to induce autistic features. The third and fourth groups were treated with low dose of either coenzyme Q (4.5 mg/kg body weight) (27) or melatonin (10 mg/kg body weight) (28) for 1 week after being intoxicated with the PA as described above (therapeutically treated groups). The fifth and sixth groups were treated with either coenzyme Q or melatonin for 1 week followed by PA intoxication (protected groups).

Rats were housed in an air-conditioned animal room and maintained at 21±1°C. They were given free access to diets and water. PA, melatonin, or coenzyme Q was orally dosed to rat pups using gastric tube.

### Tissue preparation

At the end of the feeding trials, the rats were killed by decapitation. The brains were quickly removed from the skull and dissected on ice into small pieces and homogenized in 10 times w/v bi-distilled water. Samples were stored at −80°C until the fatty acid analysis was performed.

### Ethics approval and consent

This work was approved by the Ethical Committee of Science College at King Saud University (approval no. 8/25/220358).

### Fatty acid analyses

Brain homogenate (200 µl) lipids were extracted in the presence of internal standards and fatty acid methylated using 3N methanolic HCL in sealed vials under nitrogen and incubated at 100°C for 45 min. The methyl esters of free fatty acids were extracted with hexane, and the fatty acid composition of the extract was analyzed on a gas chromatography (Hewlett-Packard 5890 series II plus, HP analytical Direct, Wilmington, DE), equipped with a flame ionization detector and a 30 m×0.25 mm×0.25 µm capillary column (Omegawax 250# 2-4136, Supelco). The helium gas flow rate was 1.2 ml/min, with a split/flow ratio of 50:1. Oven temperature was held at 205°C. The injector and detector temperatures were 260°C and 262°C, respectively. Two internal standards, C15:0 and C23:0 were added during analysis. Fatty acids were identified via comparison of retention times with authentic standards (29).

### Statistical analysis

The data were analyzed using the statistical package for the social sciences (SPSS, Chicago, IL, USA). The results were expressed as mean±standard error of the mean (SEM). All statistical comparisons between the control and PA-treated rat groups were performed using the one-way analysis of variance (ANOVA) test complemented with the Dunnett's test for multiple comparisons. Significance was assigned at the level of *P*<0.05.

## Results

Tables 1 and 2 show the alterations in the saturated and unsaturated fatty acid profiles after PA treatment and pre–post treatment with coenzyme Q10 and melatonin, respectively. The data in Table 1 shows remarkable elevation of all saturated fatty acids in the PA-treated animals compared to control, while all unsaturated fatty acids were significantly decreased in the PA-treated brain homogenates group and relatively ameliorated in the pre–post melatonin and coenzyme Q10 groups.

**Table 1:** Mean±SD together with the independent t-test for saturated fatty acid composition of brain tissue (Pg/ml) between neurointoxicated, protected, and therapeutically treated rat pups compared to healthy control

Saturated fatty acids	Control	PA	Melatonin+PPA	PA+Melatonin	CoQ+PA	PA+CoQ
Acetic	0.52±0.06	0.67±0.05	0.60±0.05	0.58±0.04	0.63±0.03	0.65±0.05
		0.001	0.013	ns	0.004	0.002
Propionic	1.23±0.16	1.97±0.36	1.63±0.09	1.51±0.10	1.56±0.11	1.64±0.06
		0.001	0.001	0.001	0.001	0.001
Butyric	0.54±0.13	0.89±0.09	0.82±0.05	0.84±0.08	0.82±0.13	0.89±0.12
		0.001	0.001	0.001	0.002	0.001
Valeric	0.13±0.02	0.22±0.02	0.21±0.02	0.21±0.02	0.21±0.03	0.24±0.03
		0.001	0.001	0.001	0.001	0.001
Hexanoic	1.73±0.20	1.96±0.12	1.84±0.17	1.85±0.12	1.74±0.13	1.79±0.19
		0.019	ns	ns	ns	ns
Caprylic	1.47±0.19	2.22±0.14	2.06±0.12	2.10±0.13	1.88±0.10	2.05±0.22
		0.001	0.001	0.001	0.001	0.001
Decanoic	1.21±0.16	1.75±0.14	1.62±0.16	1.48±0.12	1.41±0.11	1.53±0.06
		0.001	0.001	0.002	0.024	0.001
Lauric	1.01±0.21	1.65±0.12	1.63±0.15	1.84±0.14	1.80±0.12	1.80±0.12
		0.001	0.001	0.001	0.001	0.001
Myristic	1.20±0.13	1.42±0.12	1.20±0.12	1.20±0.10	1.20±0.11	1.29±0.09
		0.003	ns	ns	ns	ns
Palmitic	0.76±0.11	1.07±0.10	1.02±0.12	1.01±0.09	1.00±0.19	1.10±0.15
		0.001	0.001	0.001	0.027	0.001
Heptadecanoic	0.96±0.17	1.38±0.12	1.30±0.14	1.17±0.10	1.23±0.14	1.31±0.15
		0.001	0.001	0.010	0.007	0.001
Stearic	1.16±0.20	1.59±0.13	1.39±0.13	1.35±0.11	1.24±0.13	1.32±0.10
		0.001	0.013	0.032	ns	ns
Arachidic	0.45±0.12	0.48±0.05	0.47±0.05	0.54±0.06	0.57±0.03	0.61±0.04
		ns	ns	ns	0.019	0.005
Behenic	0.31±0.05	0.50±0.03	0.48±0.05	0.56±0.03	0.64±0.02	0.67±0.04
		0.001	0.001	0.001	0.001	0.001
Lignoceric	0.25±0.03	0.38±0.03	0.38±0.04	0.45±0.03	0.51±0.02	0.57±0.06
		0.001	0.001	0.001	0.001	0.001

**Table 2:** Mean±SD together with the independent t-test for unsaturated fatty acid composition of brain tissue (Pg/ml) between neurointoxicated, protected, and therapeutically treated rat pups compared to healthy control.

Unsaturated fatty acids	Control	PA	Melatonin+PA	PA+Melatonin	CoQ+PA	PA+CoQ
α-Linolenic	0.41±0.07	0.31±0.02	0.31±0.03	0.37±0.02	0.42±0.03	0.46±0.03
		0.004	0.002	ns	ns	ns
Stearidonic	0.20±0.04	0.45±0.04	0.41±0.04	0.38±0.03	0.40±0.03	0.46±0.04
		0.001	0.001	0.001	0.001	0.001
Linoleic	0.29±0.05	0.47±0.04	0.52±0.05	0.57±0.05	0.57±0.11	0.63±0.09
		0.001	0.001	0.001	0.001	0.001
γ-Linolenic	0.43±0.06	0.14±0.01	0.17±0.02	0.18±0.01	0.22±0.02	0.24±0.04
		0.001	0.001	0.001	0.001	0.001
Oleic	0.74±0.09	0.62±0.04	0.57±0.05	0.52±0.03	0.51±0.05	0.50±0.05
		0.004	0.001	0.001	0.001	0.001
Eicosapentaenoic	0.43±0.04	0.37±0.03	0.42±0.03	0.32±0.03	0.38±0.04	0.39±0.05
		0.006	ns	0.001	0.031	ns
Arachidonic	0.38±0.04	0.31±0.03	0.43±0.04	0.37±0.04	0.38±0.04	0.42±0.03
		0.001	ns	ns	ns	ns
Docosahexaenoic	0.64±0.07	0.53±0.05	0.64±0.06	0.51±0.04	0.52±0.08	0.54±0.09
		0.001	ns	0.001	0.006	0.029

## Discussion

This study examined the effects of pre–post treatment with coenzyme Q10 and melatonin on the fatty acid composition of brain regions in PA-induced biochemical persistent autistic features in rat pups. Saturated fatty acid levels were higher in PA-treated group when compared with normal controls, in contrast to unsaturated fatty acids. In addition, PA pre- or post-treatment with coenzyme Q10 and melatonin induced satisfactory improvement of fatty acid levels in brain tissue. PA with other saturated fatty acids, such as acetate and butyrate, are found in the gut, each of which are major metabolic products of enteric bacteria, following fermentation of dietary carbohydrates and some amino acids (30, 31). PA and its related short-chain fatty acids are capable of gaining access to the brain and inducing widespread effects on central nervous system function (32), including the impairment of neurotransmitter synthesis and release, calcium influx, intracellular pH maintenance, lipid metabolism, mitochondrial function, gap-junction dependent intercellular gating, immune activation, and gene expression, which have been proposed to contribute to the behaviors and biochemical findings observed in the PA animal model and autisms (26). In fact, compared to other fatty acids, PA was previously reported to cross the blood–brain barrier with a brain uptake index of 43.53 and a low Km value of 2.03 (33) which is enough to facilitate the penetration of PA into the brain cell, which could explain the elevation of PA in the brain homogenates of the PA treated rats. The significantly higher level of acetic acid could be related to the gastrointestinal inflammation as one of the most common clinical presentation of autism. It is well known that acetic acid–induced colitis is a well-established model (34), whereby acetate ions cause massive intracellular acidification resulting in injury of epithelial cells and inflammatory response (35).

Butyric acid is the main energy substrate for the colonocytes and is metabolized by the cells in preference to glucose or glutamine, accounting for 70% of the total energy demand of the colonic mucosa. This special fatty acid is also extensively used by the brain for the production of GABA (γ-aminobutyric acid), the natural calming agent that helps turn off stress reactions of brain. GABA does not penetrate the blood-brain barrier; it is synthesized in the brain only. In a recent study by El-Ansary et al. (36), a significant decrease in GABA was observed in rats treated with PA, which may relate to the increase in butyric acid level in the brain homogenates of the PA-treated rats in this work.

The high level of valeric acid in the PA-treated group could be related to the increase of α-keto-β-methyl valeric acid, which is a substrate for α-ketoglutarate dehydrogenase complex (mitochondrial enzyme complex for ATP synthesis via TCA cycle). In a study by Huang et al. (37), α-keto-β-methyl valeric acid was found to inhibit α-ketoglutarate dehydrogenase complex resulting in mitochondrial dysfunction and neuronal degeneration (38). Thus, α-keto-β-methyl valeric acid exposure may activate cellular events similar to those in neurodegenrative processes in PA-treated group in our study.

Stearic acid is very essential for the brain development as it is incorporated in myelin and synaptosomal lipids. Also, some of its part is actively metabolized into acetate, which in turn is used for cholesterol synthesis (39). In the current study, a high level of stearic acid was found in PA-treated group when compared with normal control, which may be the reason that this important saturated fatty acid, when taken up by the brain through blood–brain barrier, is not further metabolized and utilized in brain; the reason may be the neurotoxic effect of PA (1, 26).

Although glucose is the only fuel in the brain and fatty acids are not an energy source, mitochondria in the brain have the ability to oxidize saturated fatty acids. The β-oxidation process of the fatty acids in the brain may play a role in regulating lipid metabolism. Normally, in the brain, medium-chain fatty acids are scarcely incorporated into the lipids but mainly metabolized into glutamate and glutamine (40), whereas long-chain fatty acids such as palmitate are incorporated into the lipid fraction or became water-soluble materials, probably via the β-oxidation process (41, 42). The high levels of saturated fatty acids found in the PA-treated group in our findings may be mitochondrial dysfunction, resulting in accumulation of these acids in brain tissue (43).

Elevated levels of saturated fatty acids have been reported in the red blood cells (11, 44) and plasma (45) of several autistic patients. These findings were accompanied by a concomitant decrease in total monounsaturates, particularly, 18:1n9 fatty acid. A decline in the level of this fatty acid along with several other monounsaturates has also been observed in bloods drawn from patients with autism (44, 46, 47).

Low levels of eicosapentaenoic acid and docosahexaenoic acid were observed in the group of autistic children, which is consistent with the evidence of altered lipid metabolism in neuropsychiatric disorders (48). It has been proposed that such imbalance is related to changes in the structure and function of cell membrane phospholipids. In particular, the levels of eicosapentaenoic acid have been associated with the production of eicosanoids, which have anti-inflammatory, antithrombotic, and vasodilator properties (15).

PA infusion resulted in decreased levels of total monounsaturates and total omega 6 (n-6) fatty acids (48). Omega-3 supplementation has been associated with better results in behavioral assessment scales applied to autistic children (49). In recent research, there is growing interest on the potential roles of the omega-3 polyunsaturated fatty acid docosahexaenoic acid and precursor eicosapentaenoic acid with regard to the brain structure, function, and mental health in human beings (50, 51). In fact, docosahexaenoic acid is particularly concentrated at neural synapses, sites of neurotransmitter signaling. Omega-6 PUFA arachidonic acid is also abundant in the brain, reflecting a key role in brain structure and function. Linoleic, an essential fatty acid, is used in the biosynthesis of arachidonic acid. Abnormalities in fatty acid metabolism may result in a decreased level of arachidonic acid with an increase in linoleic (52). This may be the reason for low level of arachidonic acid and high level of linoleic in the PA-treated group. Likewise, the arachidonic acid precursor, γ-linolenic acid, and docosahexaenoic acid precursor, eicosapentaenoic acid, are all considered to play key roles in brain functioning, especially via the synthesis of eicosanoids (51).

Based on the observation of the present study, the recorded depletion for most polyunsaturated fatty acid could perhaps be attributed to the brain abnormalities in PA-intoxicated rats and suggests that dietary supplementation with high polyunsaturated fatty acid could be suggested as a treatment strategy. This suggestion is consistent with the recent work of El-Ansary et al. (53), which proved the potential protective effect of omega-3 against PA-induced neurotoxicity. The stearidonic acid concentration tends to be low normally because it is formed slowly by the desaturation of α-linolenic, catalyzed by δ-6-desaturase, and is then quickly elongated to other metabolites (54). High level of stearidonic acid in all PA-treated groups may again be due to neurotoxicity, resulting in the improper metabolism of stearidonic acid, as abnormalities in the metabolism of essential fatty acid or/and their long-chain polyunsaturated metabolites are often reported in autism (15).

Melatonin and coenzyme Q10 restore the fatty acid levels in brain tissue of PA-treated rats to levels proximate to those in healthy control rats as shown in Tables 1 and 2. Melatonin has multiple actions as a regulator of antioxidant enzymes, radical scavenger, and antagonist of mitochondrial radical formation. Melatonin has the ability to interact directly with the electron transport chain by increasing the electron flow and reducing electron leakage, thus increasing the survival of neurons under enhanced oxidative stress (55). Coenzyme Q10 is responsible for energy generation through the mitochondrial respiratory chain and increases fatty acid oxidation through the AMP-activated protein kinase (AMPK) pathway (56). AMPK is an important regulator of energy balance. AMPK stimulates catabolic pathways, including glucose and fatty acid oxidation (57–59), while simultaneously reducing anabolic pathways (cholesterol, fatty acid, and triacylglycerol synthesis) (60). Various studies indicate that the neurotoxicity related to mitochondrial dysfunction may be ameliorated by coenzyme Q10 (61).

## Conclusions

To sum up, though this study shows evidence of the efficacy of melatonin and coenzyme Q in ameliorating most of the impaired fatty acid profile in rodent model with persistent autistic features, there is a need for better designed and registered trials of at least 6 months in length, in order to reliably identify a confirmed proper effect.
